# *Staphylococcus aureus* ST398, New York City and Dominican Republic

**DOI:** 10.3201/eid1502.080609

**Published:** 2009-02

**Authors:** Meera Bhat, Caroline Dumortier, Barbara S. Taylor, Maureen Miller, Glenny Vasquez, Jose Yunen, Karen Brudney, Jacqueline Sánchez E., Carlos Rodriguez-Taveras, Rita Rojas, Patricia Leon, Franklin D. Lowy

**Affiliations:** Columbia University, New York, NY, USA (M. Bhat, C. Dumortier, B. Taylor, G. Vasquez, K. Brudney, F.D. Lowy); New York Medical College, Valhalla, NY, USA (M. Miller); Centro de Diagnostico y Medicina Avanzada y de Conferencias Medicas y Telemedicina, Santo Domingo, Dominican Republic (J. Yunen); Laboratorio Referencia Clinico, Santo Domingo (J. Sánchez E., P. Leon); Hospital Central de Las Fuerzas Armadas, Santo Domingo (C. Rodriguez-Taveras); Centro Medico Luperon, Santo Domingo (R. Rojas)

**Keywords:** Staphylococcus aureus, colonization, transmission, emigration and immigration, animal exposure, dispatch

## Abstract

Closely related *Staphylococcus aureus* strains of ST398, an animal-associated strain, were identified in samples collected from humans in northern Manhattan, New York, NY, USA, and in the Dominican Republic. A large population in northern Manhattan has close ties to the Dominican Republic, suggesting international transmission.

In the past 5 years, as methicillin-resistant *Staphylococcus aureus* (MRSA) has emerged as a community pathogen, awareness of the role of animal exposure from pets or farming as sources of MRSA has increased (*1*–*3*). We identified a clone of *S. aureus* previously associated with outbreaks of infections in animals and in humans who work with animals in 2 unique collections of *S. aureus* isolates. The first was from a population-based study of *S. aureus* colonization among residents of northern Manhattan in New York, NY, USA; the second was from isolates obtained from the Dominican Republic. This clone does not digest with the restriction enzyme *Sma*I, which is generally used for pulsed-field gel electrophoresis (PFGE). Consequently, the clone is identified by multilocus sequence typing as sequence type 398 (ST398). Both methicillin-resistant and methicillin-susceptible isolates of *S. aureus* have been reported (*4*). ST398 has been found primarily in Europe, where it has been isolated from pigs and pig farmers in the Netherlands and France and from dogs, pigs, horses, and humans in Germany and Austria (*5**–**8*). Colonization with MRSA ST398 has recently been reported in pigs and pig farmers in Canada (*9*).

## The Study

The community-based study was conducted from 2004 through 2007 in the northern section of Manhattan, a borough of New York City. Northern Manhattan contains a large, medically underserved population that has close ties to the Dominican Republic. Participants were recruited by using random-digit dialing. Consenting persons and household members were subsequently interviewed and screened for *S. aureus* colonization. A total of 321 eligible households containing 914 household members participated. In 9 households, 13 participants were found to be colonized with *S. aureus* isolates that were *Sma*I resistant. Digestion with the *Cfr9*I, an isoschizomer of *Sma*I, yielded identical PFGE profiles (Figure). Subsequent multilocus sequence typing confirmed the ST398 identification (allelic profile 3–35–19–2-20–26–39). All strains were methicillin susceptible. A representative strain was *spa*-typed as type t571 (allelic profile 8–16–2-25–2-25–34–25, eGenomics type 109); it was Panton-Valentine leukocidin negative.

**Figure:**
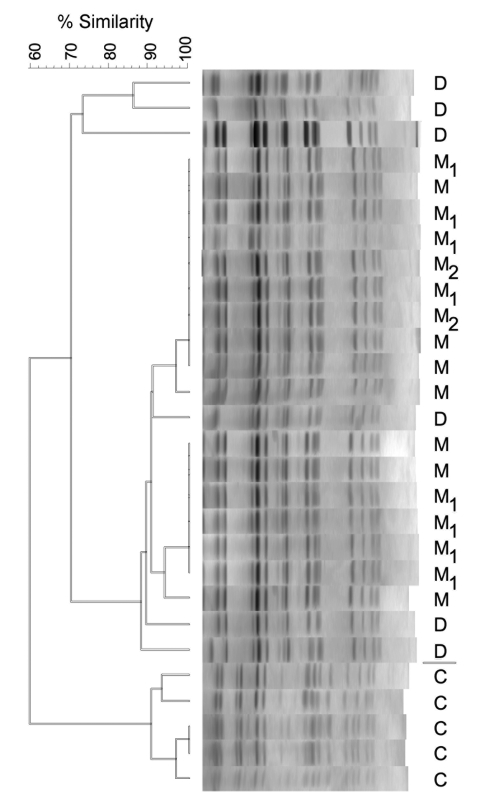
Pulsed-field gel electrophoresis profiles of sequence type 398 isolates from the Dominican Republic (D); northern Manhattan (M), New York, NY, USA; and Canada (C) (provided by Scott Weese). Strains within households in which >1 person was colonized are identified numerically. The dendrogram shows the percent similarity of the isolates. A similarity >70% indicates closely related or identical strains.

Characteristics of persons colonized with ST398 were similar to those of persons in the community-based study and with northern Manhattan census characteristics (Table). The 13 isolates were from 9 different families; 1 family had 4 members colonized with ST398 at either nasal or axillary sites. The mean age of those colonized was 33.4 years; only 1 child (7 years of age) was colonized. Two persons from different families were colonized with ST398 at multiple sites, none of which were confirmed as infections.

**Table:** Characteristics of persons colonized with *Staphylococcus aureus* ST398, northern Manhattan, New York City, NY, USA, 2004–2007, compared with study population and 2000 census population for area*

Characteristics	ST398 subset, no. (%)	Overall study population, no. (%)	2000 census population, no. (%)
Race/ethnicity			
Latino	11 (84.6)	813 (89)	173,755 (68)
Non-Hispanic white	2 (15.4)	90 (9.8)	65,449 (25.6)
African American	0	11 (1.2)	53,514 (20.9)
Asian	0	0	5,370 (2.1)
Sex			
Male	4 (30.8)	362 (39.6)	120,866 (47.3)
Female	9 (69.2)	552 (60.4)	134,723 (52.7)
Age group, y			
<5	0	84 (9.2)	17,878 (7.0)
5–17	1 (7.6)	238 (26.2)	49,196 (19.3)
18–44	10 (76.9)	297 (32.5)	112,195 (44.0)
>45	2 (15.4%)	290 (31.9)	76,320 (29.9)
Occupational exposure	5 (38.5)	58 (6.4)	NA
Travel outside USA	7 (53.8)	171 (18.7)	NA
Daycare exposure	2 (15.4)	87 (9.5)	NA
Total population	13 (100)	914 (100)	255,589 (100)

*ST, sequence type; NA, not available.

No household reported owning pets, although 2 reported animal contact. Of the 12 adults, 5 (41.7%) reported possible job exposure to *S. aureus*, including 1 who worked in a healthcare-associated field. No household reported patronizing *viveros*, or live poultry markets, which are common in the Latino communities of northern Manhattan and the Bronx. Two households reported having children who attended day care, although none of these children were colonized with *S. aureus*. Although 15% of the Dominican population in the study reported travel to the Dominican Republic within 6 months of their interview, none of the colonized participants reported recent travel to the Dominican Republic. No contact among the different households was reported.

A second collection of *S. aureus* isolates was gathered during 2007 and 2008 from a convenience sample of 89 anonymous infection and colonization isolates received from the Dominican Republic. Six isolates were identified as methicillin-susceptible *S. aureus* clone ST398. Strains were provided by 1 hospital in Santo Domingo, Dominican Republic (n = 53), and 1 microbiology laboratory, the Laboratorio Referencia (n = 28), which serves as a reference laboratory for the country. Four isolates from the hospital and 2 from Laboratoria Referencia were identified as methicillin-susceptible ST398. Sociodemographic data on these persons were limited. Two of the ST398 isolates were from women with infections living in Santo Domingo, and the remaining 4 were colonization samples. Of the 6 isolates, 5 were found to be *spa*-type t571 (eGenomics type 109), and 1 was found to be type t3625 (eGenomics untyped).

Pairwise similarity scores for the isolates were calculated by the Dice coefficient, and an overall similarity score was calculated by using the unweighted pair group method with arithmetic mean. Comparing the isolates by using a dendrogram-based similarity score >70%, we found that the strains from northern Manhattan and from the Dominican Republic were closely related, although they contrasted with ST398 isolates from Canada (provided by Scott Weese, Ontario Veterinary College, University of Guelph) (Figure) (*10*).

## Conclusions

Identification of the *S. aureus* clone ST398 in northern Manhattan and in the Dominican Republic suggests its introduction into the United States by travelers between the 2 countries. The largely Latino population of northern Manhattan is composed mainly of immigrants or first-generation families from the Dominican Republic; travel between the 2 regions is common. Alternatively, northern Manhattan may contain reservoirs, such as live poultry markets, which may serve as a means of strain transmission.

Colonization or infection with the *S. aureus* clone ST398 has been associated with exposure to pigs, pets, and other animals (*5**,**7*–*9*), and the *S. aureus* clone ST398 has been isolated from meat products (*2*). However, transmission is not limited to animal exposures. Person-to-person spread has occurred among household members and in the hospital setting (*6**,**8**,**11*). For example, a dramatic increase in persons colonized as well as infected with MRSA clone ST398 was recently reported in a Dutch hospital (*12*).

The presence of this strain among several household members in our study reinforces earlier observations of the potential for horizontal transmission of this clone after it is introduced into an appropriate setting. Although information about the persons from the Dominican Republic was limited, the 2 groups provided identical strain profiles, suggesting a possible link between the 2 countries. Given ST398’s history of rapid dissemination in the Netherlands, its potential for the acquisition of methicillin resistance, and its ability to cause infections in both community and hospital settings, monitoring the prevalence of this strain in northern Manhattan and the Dominican Republic will be important to understand more about its virulence and its ability to spread in these communities.
